# Physical Fitness and the Healing of Wounds

**Published:** 1914-10-03

**Authors:** 


### The Hospital

**The Workers' Newspaper of Administrative Medicine and Institutional Life, Administration, National Insurance and Health.**

No. 1476, Vol. LVII. SATURDAY, OCTOBER 3, 1914. PRICE ONE PENNY.

LIBRARY
230053

### PHYSICAL FITNESS AND THE HEALING OF WOUNDS.

The medical aspects of the war now in progress on the Continent are numerous, and in due course of time the occasion will arise for their ordered presentation and study. From this occasion, it is to be feared, we are still far removed, but in the meantime the material necessary for its effective use is gradually accumulating. Obviously, to take but a single point, the war will offer on a most extensive scale opportunities for the study of the healing of various forms of wounds. Such a study has both a pathological, and a military and economic interest. On the pathological side it bears particularly on the effects produced by the several kinds of weapons employed by armies in the field and on the promotion of the processes of healing; whilst the extent to which men are disabled by wounds and the period of time during which such disablement persists, are considerations of high importance to those responsible for the organisation and financing of a great army.

Anything like a comprehensive judgment on the issues we have just raised is manifestly at present impossible, and it would be absurd to offer a considered verdict. There are, however, certain facts which have begun to attract attention in relation to the experience of those responsible for the surgical care of the wounded men now lodged in hospitals in this country. First there is the observation that in a very large proportion of instances the wounds received in battle tend to heal readily and satisfactorily. Doubtless there are individual exceptions, but the general rule is as above stated. The conclusion is a very satisfactory one both for the individual immediately concerned and for the country. For the former it means a good recovery, and for the latter, at least in many instances, such recovery restores an efficient soldier to the fighting line.

There are in operation several factors which tend to promote the rapid healing of the wounds inflicted in modern battles, as contrasted with the hospital horrors of an earlier day. Probably the chief of these is the arrangement which secures the early application of an antiseptic dressing to the wound. In this way the risk of septic complications is very considerably diminished and the miseries of suppuration are obviated. Lister's work has had a far-reaching effect, and amongst those who are his debtors must be numbered soldiers of all nations who fall on the stricken field. In the second place there is, of course, a great difference between the damage done by a narrow, swift-travelling bullet and that inflicted by a missile which crushed and bruised the tissues rather than penetrated them. The South African War taught us that many penetrating wounds, even of the abdomen and thorax, were capable of healing under the kindly influence of Nature and without interference from the art of the surgeon. Similar experiences are already being noted in the present contest.

A third influence which doubtless bears on the rapid healing of gunshot and other wounds is the tissue capacity of the wounded man, as this is determined by his general health and physical vigour. Whether the *vis medicatrix naturae* is quite so benevolent as is often suggested may be doubted, but there is no question of her ability and force. The healing processes come from within, and unless they exist, at least potentially, manipulations and operations and applications are all alike in vain. Now there seems some reason to believe—judged by the fashion in which their wounds heal—that our soldiers possess in a high degree that form of vigour which promotes the rapid restitution of damaged tissues and the re-establishment of the balance of health. It is impossible to avoid the conviction that such a state of matters is largely due to the culture of the open-air life which is so popular with the young manhood of our country. Cricket, football, and the like are just now—and perhaps quite rightly—under the popular frown, but they have served other ends than mere amusement; and for our own part we should not be sorry to see our new recruits getting opportunities once or twice a week for a vigorous contest between the goals. Drill and other military exercises must have the first place, of course, and the systematic organisation of the military machine is essential. At the same time in the rough work of war we

W1
H069X

cannot overvalue the fitness of the individual soldier, his ability to endure, and his capacity to seize or make an opportunity. Add to these ends the further consideration that the discipline which produces them promotes also the tissue health which is necessary for the rapid healing of wounds, and the argument for the outdoor life and for open-air games need not be further emphasised.

